# Role of elastic fiber degradation in disease pathogenesis

**DOI:** 10.1016/j.bbadis.2023.166706

**Published:** 2023-03-29

**Authors:** Gregory Halsey, Dipasha Sinha, Saphala Dhital, Xiaoying Wang, Naren Vyavahare

**Affiliations:** Department of Bioengineering, Clemson University, SC 29634, United States of America

**Keywords:** Elastin, Extracellular matrix degradation, Elastin derived peptides, Cellular signaling

## Abstract

Elastin is a crucial extracellular matrix protein that provides structural integrity to tissues. Crosslinked elastin and associated microfibrils, named elastic fiber, contribute to biomechanics by providing the elasticity required for proper function. During aging and disease, elastic fiber can be progressively degraded and since there is little elastin synthesis in adults, degraded elastic fiber is not regenerated. There is substantial evidence linking loss or damage of elastic fibers to the clinical manifestation and pathogenesis of a variety of diseases. Disruption of elastic fiber networks by hereditary mutations, aging, or pathogenic stimuli results in systemic ailments associated with the production of elastin degradation products, inflammatory responses, and abnormal physiology. Due to its longevity, unique mechanical properties, and widespread distribution in the body, elastic fiber plays a central role in homeostasis of various physiological systems. While pathogenesis related to elastic fiber degradation has been more thoroughly studied in elastic fiber rich tissues such as the vasculature and the lungs, even tissues containing relatively small quantities of elastic fibers such as the eyes or joints may be severely impacted by elastin degradation. Elastic fiber degradation is a common observation in certain hereditary, age, and specific risk factor exposure induced diseases representing a converging point of pathological clinical phenotypes which may also help explain the appearance of co-morbidities. In this review, we will first cover the role of elastic fiber degradation in the manifestation of hereditary diseases then individually explore the structural role and degradation effects of elastic fibers in various tissues and organ systems. Overall, stabilizing elastic fiber structures and repairing lost elastin may be effective strategies to reverse the effects of these diseases.

## 1. Introduction

Elastin is an extracellular matrix (ECM) protein critical to the structural integrity of tissues, including arteries, lungs, and skin. Crosslinked elastin and associated microfibrils, named elastic fiber, contribute to biomechanics by providing the elasticity required for proper function. Understanding elastic fiber assembly is essential to comprehending the consequences of elastin degradation in diseases.

**Fig. 1.**
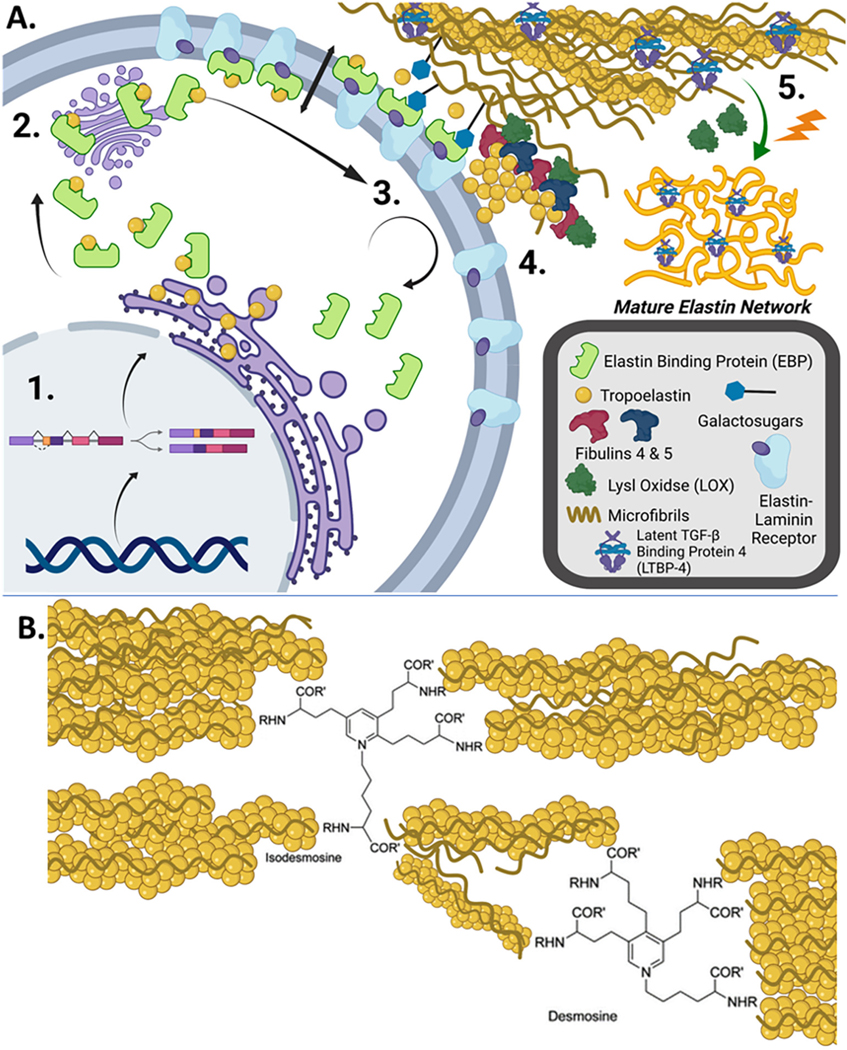
Elastin synthesis and crosslinking. A) Illustration of mature elastin fiber synthesis: (1) Tropoelastin (TE) mRNA is synthesized from the Eln gene, spliced, then translated and bound to EBP at the surface of the rough endoplasmic reticulum. (2) EBP-bound tropoelastin is transported through the Golgi Apparatus to the cell membrane (3) tropoelastin is released by EBP outside the cell forming amorphous chains while EBP is reused inside the cell. (4) Fibulins align tropoelastin and bind LOX which catalyzes the formation of covalent intra- and intermolecular crosslinks between lysine residues until the amorphous elastin reaches a critical size. Crosslinked elastin detaches from the plasma membrane and aggregates onto microfibrils along with latent TGF-β / LTBPs. (5) Fibrils are crosslinked further by LOX to form mature elastin network. EBP: Elastin Binding Protein; TE: Tropoelastin LOX: Lysl Oxidase, B. One lysine and three allysines crosslink into desmosine or isodesmosine to create crosslinked insoluble elastin fibers extracellularly.

Endothelial cells, smooth muscle cells, fibroblasts, and myofibroblasts synthesize the precursor to elastin, tropoelastin (60–70 kDa) [1]. During transfer into the rough endoplasmic reticulum, its 26 amino acid N-terminal signal sequence is cleaved. Once within the RER lumen, tropoelastin binds to elastin binding protein (EBP) and is then transported to the Golgi apparatus and subsequently the cell membrane [1–3]. Galacto-sugars from extracellular glycoproteins such as fibulins bind to the galactolectin site of EBP, causing tropoelastin release and secretion to the extracellular matrix. EBP is recovered by the cell to be reused while tropoelastin monomers are aligned within the glycoprotein scaffolds as they integrate to extend the elastic fiber [3]. Fibulins 4 and 5 contribute to the formation of mature elastin by binding to tropoelastin, fibrillin-1, and copper-dependent enzyme lysyl oxidase (LOX) to aid structural assembly as well as activating LOX (Fig. 1) [4–6]. The LOX enzyme creates hydroxylysine residues on tropoelastin molecules. Multiple tropoelastin molecules are then crosslinked in desmosine and isodesmosine units to create crosslinked mature elastic fiber (Fig. 1) [7].

Mature elastic fibers consist of an insoluble crosslinked elastin center surrounded by microfibrils primarily composed of fibrillin, which bind several proteins involved in fiber formation including: microfibril-associated glycoproteins, microfibrillar-associated proteins, and fibulins. Elastin biosynthesis starts in the womb and peaks just before birth but decreases throughout childhood and begins to stop during puberty; the half-life of elastin is around 70 years, and it rarely remodels in adults [8,9]. Microfibrils make up only around 15 % of the elastic fibers in adults but account for approximately 50 % in children [7]. An additional component of mature elastic networks is latent transforming growth factor β (TGF-β) binding proteins (LTBPs). LTBPs bind to latent TGF-β and are structurally related to fibrillins, facilitating localization to microfibrils and sequestration within elastic fiber networks during formation [10]. LTBP remains within the mature elastic network containing inactive latent TGF-β until released and activated by either mechanical force or proteolytic cleavage [10].

## 2. Role of elastin-derived peptide fragments (EDPs) in cellular behavior and systemic pathology

Due to limited synthesis, damage to elastic fibers with aging or disease remains unrepaired throughout an individual’s lifetime. During inflammatory disease conditions, elastic fiber can be degraded by proteolytic enzymes known as elastases, including proteinases from the serine, cysteine, and metalloproteinase families. Enzymes such as neutrophil elastase, proteinase 3, G, L, S, K, and V-cathepsin as well as matrix metalloproteinases (MMPs) −2, 7, 9, and 12 degrade elastin [11–14]. It has been observed *in vivo* that human macrophages adjacent to fragmented elastin express MMP-7, 9, and 12 [15]. In the absence of plasminogen, human macrophages predominantly secrete MMP-12, which binds directly to elastin. While in presence of plasminogen, these macrophages activate MMP-7 and drastically increase proteolysis [15]. Elastin degradation creates EDPs with a bioactive VIII β-turn conformation GxxPG motif that binds to the EBP subunit on cells [13]. EDPs have been identified as ligands for galectin-3, αVβ3 and αVβ5 integrins, lactose insensitive receptors, as well as EBP [16–18]. EDP binding to EBP activates Neu-1, converting GM3 ganglioside I to lactosylceramide, which acts as a secondary messenger [19]. Moreover, Neu-1 is an important regulator of several receptors, including insulin receptor, hepatocyte growth factor receptor, insulin growth factor receptor, platelet derived growth factor receptor, and several Toll-like Receptors [20–22].

**Fig. 2.**
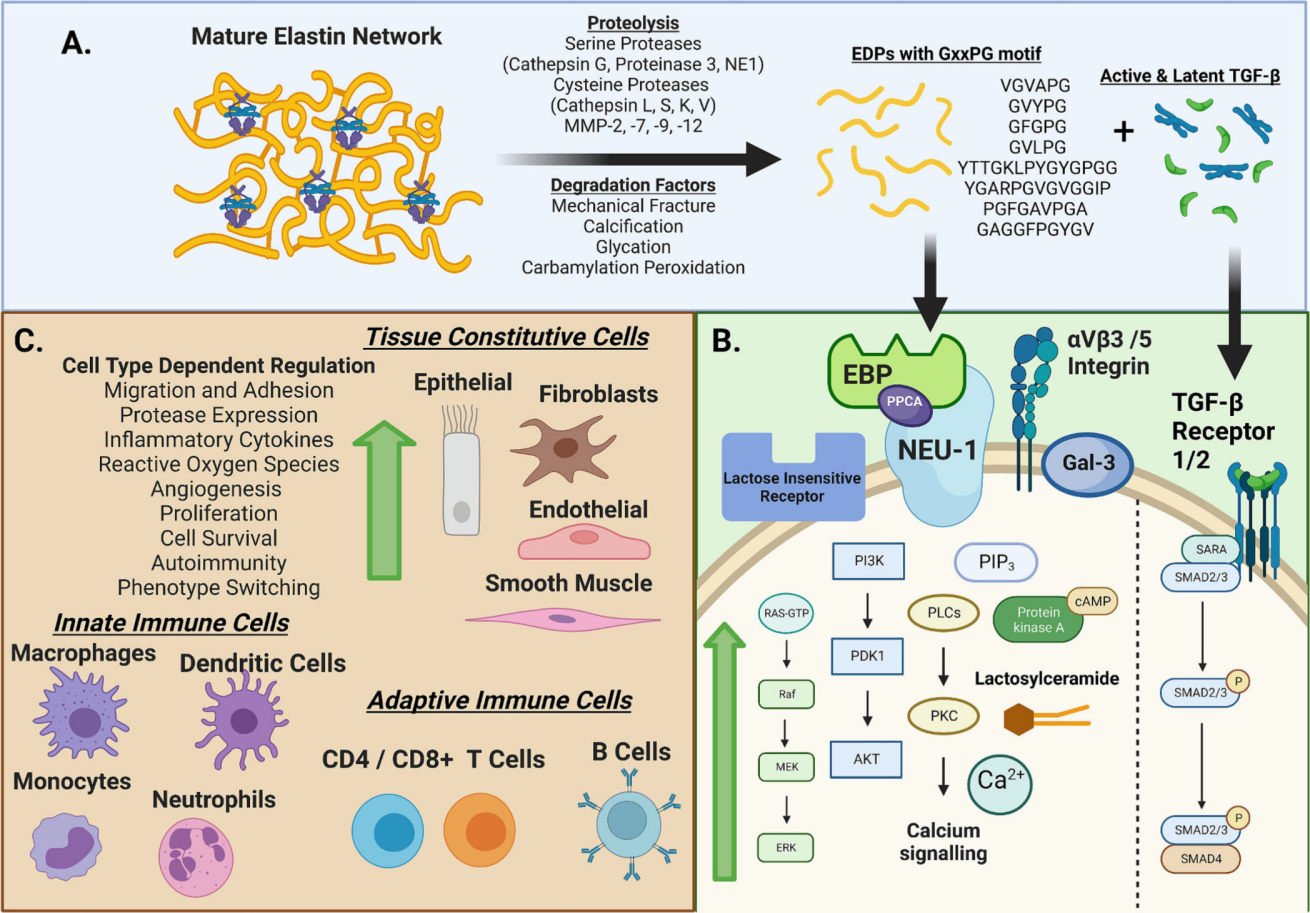
Illustration demonstrating elastic fiber degradation and cellular effects induced by elastin-derived peptides (EDPs). EDPs and TGF-β are released by mechanical, chemical, or enzymatic damage to elastin network. EDPs bind to lactose insensitive receptor, EBP, galectin-3, as well as αVβ3 and αVβ5 – triggering subsequent downstream signaling associated with increased calcium and lactosylceramide. Cellular phenotype effects are observed in a wide variety of cells including tissue constitutive cells that comprise tissue architecture as well as innate and adaptive immune cells. The effects of EDPs are primarily inflammatory and cause loss of homeostatic cell status resulting in disruption of tissue structure and function by degradative remodeling. NEU-1: Neuraminidase-1, EBP: Elastin Binding Protein, Gal-3: Galectin-3

EDPs affect many signaling pathways as summarized in Fig. 2 Panel B. In arterial vascular smooth muscle cells (VSMCs), the elastin receptor complex activates G proteins, L-type calcium channels, focal adhesion kinase, c-Src kinase, platelet derived growth factor receptor kinase, as well as Ras-Raf-MEK1/2-ERK1/2, subsequently stimulating proliferation [21,23]. In fibroblasts, EDPs activate the MEK1/2 and ERK1/2 pathways *via* protein kinase A and phosphoinositide 3-kinase γ [24]. In endothelial cells, EDPs stimulate phosphoinositide 3-kinase γ and Akt to coordinate with endothelial nitric oxide synthase, nitric oxide, and protein kinase G activating ERK1/2; additionally, protein kinase C signaling activation is observed [25]. EDPs cause intracellular calcium flux *via* the PLCγ pathway leading to the production of IP3 primarily in neutrophils and monocytes [26–28].

EDPs exhibit diverse biological effects which are summarized in Fig. 2 Panel C. EDPs are chemotactic to endothelial cells, monocytes, neutrophils, macrophages, fibroblasts, and VSMCs as well as promote osteogenesis and reactive oxygen species generation in VSMCs and fibroblasts [29–31]. EDPs were also shown to induce epithelial/endothelial to mesenchymal transition, a process by which cells lose characteristics of differentiated cells and exhibit mesenchymal-like phenotypes [32]. Coinciding with this, EDPs have angiogenic effects observed to influence neovascularization in cancer, ophthalmic disease, and atherosclerosis [33,34]. EDPs affect immune cell phenotypes, promoting M1 macrophage polarization and Th1/Th17 differentiation, increasing local inflammation [35,36]. Exposure to EDPs also generates a specific adaptive immune response, creating auto-reactive T and B lymphocytes as well as antibodies in smokers and patients with asthma, emphysema, chronic obstructive pulmonary disease, systemic sclerosis, thoracic aortic aneurysms (AAs), as well as other diseases related to elastin degradation [37–40].

The modification of cellular behavior by EDPs often enhances pathogenesis and accelerates disease progression *in vivo*. Sensitization of mice with EDPs predisposes them to more severe elastase induced aneurysm growth associated with autoimmunity and expansion of Th17 cells [41]. Similarly, immunization of mice with EDPs exacerbated smoke-induced ocular pathology and vision loss which was further increased when EDPs were oxidized by cigarette smoke prior to administration [42]. EDPs also inhibit glucose tolerance in mice and promote insulin resistance through interaction with the elastin receptor complex, specifically neuraminidase 1, and insulin receptors, forming a positive feedback loop accelerating diabetes and complications [20]. EDP exposure increases the size of atherosclerotic plaques at the aortic root, and rabbits receiving intravenous injections of EDPs show elevated serum elastase activity, developing atherosclerosis independent of other stimuli [43,44]. EDPs can also accelerate the pathogenesis of liver disease in non-alcoholic fatty liver disease and non-alcoholic steatohepatitis by increasing the accumulation of hepatic triglycerides, type I collagen, tropoelastin, fibulin-5, and fibrillin-1. Elastin receptor complex/neuraminidase 1 activation by EDPs reduce hepatocyte growth factor receptor activity by cleaving N-glycosylation chain sialic acids, increasing lipotoxicity, oxidative stress, and inflammation initiating the transition from non-alcoholic fatty liver disease to non-alcoholic steatohepatitis [22].

Elastic fiber degradation also causes cleavage of microfibril bound LTBPs and release of soluble latent and active TGF-β; subsequently, MMPs such as 2 and 9 that degrade elastin components of elastic fibers also activate latent TGF-β [45–47]. Canonical TGF-β signaling, as illustrated in Panel B of Fig. 2, contributes to effects observed in elastin-related diseases. TGF-β activity increases migration and proliferation of VSMCs, fibroblast contraction, deposition of collagen, and epithelial/endothelial to mesenchymal transition processes [46,47]. The coinciding release of EDPs and TGF-β may accentuate pathogenesis; however, there is also evidence suggesting that TGF-β exerts protective effects known as the “TGF-β Paradox,” which is evident in cancer and vasculopathy. This review will cover the role of elastin degradation in the establishment and progression of various diseases in different organ systems.

## 3. Elastin degradation related hereditary diseases

**Fig. 3.**
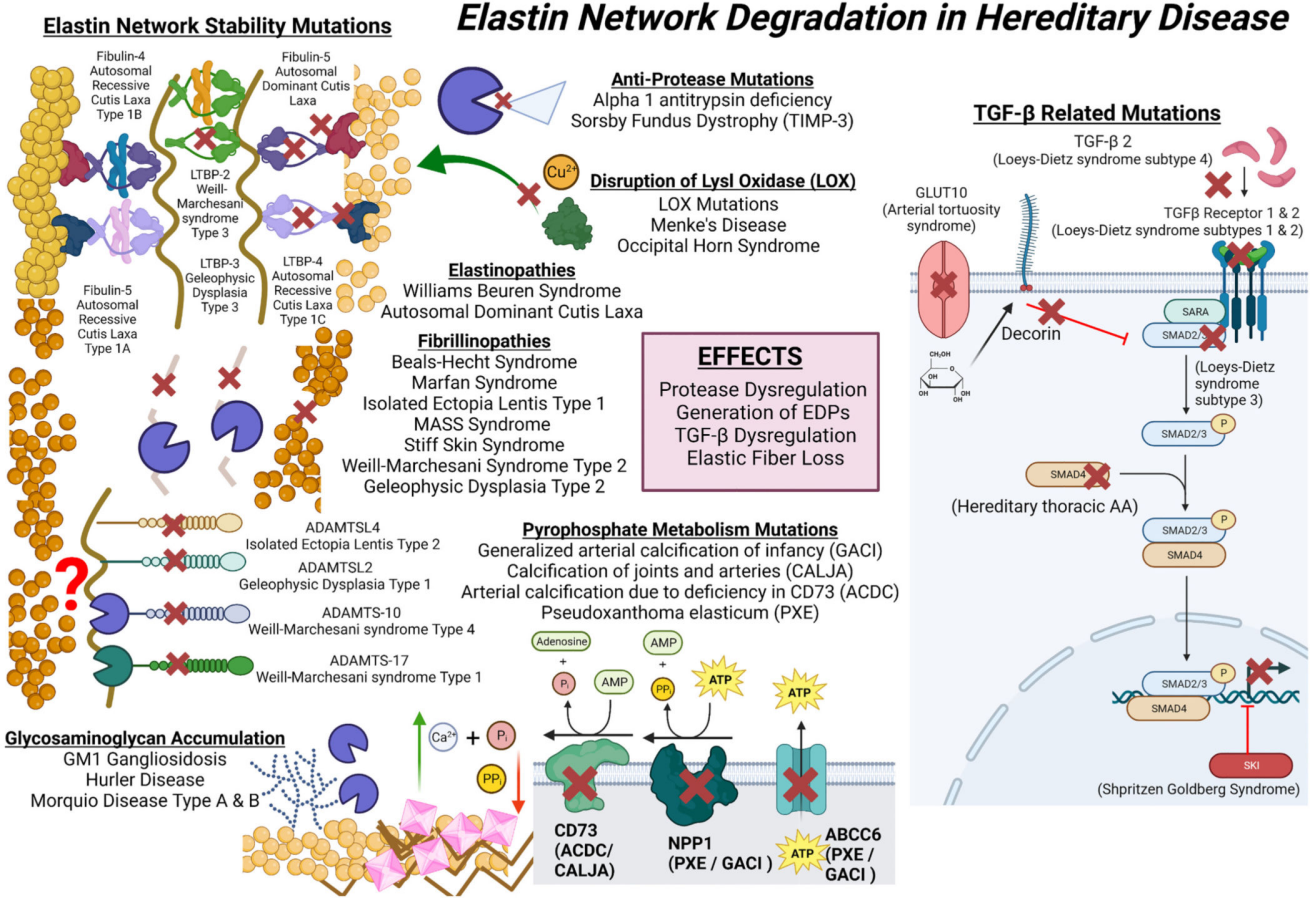
Illustration demonstrating molecular basis for various hereditary diseases discussed related to elastin degradation. Elastic fibers which are irregular due to reduced or modified tropoelastin and fibrillin-1 are prone to enzymatic degradation and release EDPs / TGF-β causing feedback resulting in further damage. Loss of LOX activity by direct mutation or reduced copper availability results in accelerated elastin matrix degradation and manifestations of disease. Disruption of pyrophosphate homeostasis causes elevated inorganic phosphate which accelerates degradation of elastin fibers and induces calcium phosphate crystal deposition.

As summarized in Table 1 and Fig. 3, genetic defects that impact elastic networks directly or indirectly highlight the potential pathological consequences of elastin degradation. Conditions result from mutations of the elastin gene (elastinopathies), the microfibrillar scaffolding (fibrillinopathies), proteins involved in maintenance of elastic/ECM networks, proteins that regulate crosslinking and degradation, as well as elements of the TGF-β signaling pathway.

### 3.1. Elastinopathies

Mutations of the tropoelastin gene results in systemic manifestations of disease which are particularly noticeable in the skin. Williams Beuren Syndrome patient skin has fewer, fragmented elastic fibers with decreased resistance to degradation [82]. On a molecular level, Williams Beuren patient skin elastic fibers were found to have reduced hydroxyproline content, correlated with susceptibility to enzymatic degradation [83]. Interestingly while these properties affect the biomechanics of the skin and are statistically different from age matched controls, there was no correlation between dermal and vascular defects, suggesting tissue specific regulation of elastic matrix homeostasis [84]. In autosomal dominant cutis laxa, dermal elastin is also degraded, with presentation typically being more severe than Williams Beuren Syndrome. Expression of autosomal dominant cutis laxa mutant tropoelastin in fibroblasts results in greatly reduced deposition of tropoelastin onto microfibril structures, with tropoelastin preferentially forming immature aggregates. These fibroblasts also show increased phosphorylation of SMAD2, endoplasmic reticulum stress, and apoptosis indicating TGF-β signaling is dysregulated [51].

While dermal presentations are mild, vascular complications in Williams Beuren Syndrome can be severe. Reduction of elastin synthesis by VSMCs in Williams Beuren Syndrome and supravalvular aortic stenosis is associated with dysregulation of MMP-9/tissue inhibitor of metalloproteinase-1 (TIMP-1) leading to enhanced elastolytic capability [85]. Similarly, in a study of 8 autosomal dominant cutis laxa patients, serum MMP-2 and 9 were significantly upregulated compared to normal levels suggesting systemic elastin degradation in autosomal dominant cutis laxa is due to protease dysregulation [86]. Seemingly contradictory, while both *Eln* +/− mice and William’s Beuren Syndrome have reduced total elastin content, there is an increased number of elastic lamellae observed outside regions of stenosis [87]. At lower pressures, *Eln* +/− mice have a significant reduction in aortic circumferential stiffness with some features of premature cardiovascular aging such as reduced vascular elasticity, fragmented elastic fibers, and intimal fibrosis [88]. Interestingly, the lifespan of *Eln* +/− mice does not differ significantly from wild type and *Eln* +/− mice were protected from arterial thickening observed in aging of wild type mice. There was no difference in desmosine and hydroxyproline content of the ascending aorta in *Eln* +/+ and *Eln* +/− mice after aging, however, both young and aged *Eln* +/− mice had greatly reduced desmosine content compared to wild type [89]. Mirroring results seen in *Eln* +/− mice, Williams Beuren patient aortic elastic fibers showed no difference in hydroxyproline content as compared to unaffected individuals. However, in contrast with *Eln* +/− mice, Williams Beuren patient aortic elastin desmosine/isodesmosine content does not differ from healthy aortic elastin [83]. Since elastin network synthesis ceases in adults, improper establishment of elastin networks due to elastinopathies may particularly predispose them to degradation by mechanisms observed during natural aging processes or after exposure to harmful/pathogenic stimuli resulting in disease. Furthermore, William’s Beuren Syndrome and autosomal dominant cutis laxa, VSMCs have a greater proliferative capacity with reduced elastin secretion and deposition [81,90]. Disorganization and stiffness of the ECM substrate is correlated to VSMC dedifferentiation, proliferation, and migration thus providing another mechanism linking development of stenotic disease in elastinopathies to elastic fiber disruption [91,92].

It is unclear why tropoelastin mutations in Williams Beuren Syndrome induce mild dermal disease and severe cardiovascular disease while autosomal dominant cutis laxa is associated with drastic dermal alterations and pulmonary disease. Given that Williams Beuren Syndrome chiefly reduces tropoelastin synthesis and autosomal dominant cutis laxa mutations produce altered tropoelastin protein, this is likely a key factor in the differential manifestation of elastin related disease. Mutated tropoelastin associated with severe pulmonary autosomal dominant cutis laxa phenotypes was improperly secreted and particularly prone to intracellular accumulation suggesting this may effect development of pathology [90]. Reduced secretion of tropoelastin is shared between elastinopathies, however, gain-of-function effects such as intracellular accumulation seen in autosomal dominant cutis laxa may participate in tissue specific pathogenesis.

### 3.2. Fibrillinopathies

Mutations in the fibrillin genes (fibrillinopathies) cause a reduction, modification, or disorganization of microfibrils. Disruption of fibrillin-1 in Marfan syndrome patients is correlated to decreased expression of aortic TIMP-3 with increased expression of MMP-2, 9 and 12. Subsequently, soluble aortic extracts from Marfan syndrome patients and mice models induce macrophage chemotaxis, which can be neutralized by pre-treatment with anti-elastin (VGVAPG) antibody, lactose (EBP antagonist), or EDPs implying involvement of the EBP and EDPs in inflammatory processes [93]. Evidence suggests fibrillin mutations in Marfan syndrome produce unstable elastic fibers prone to degradation which release EDPs and fibrillin fragments containing the XGXXPG sequence, stimulating monocyte/macrophage chemotaxis, infiltration, and inflammatory polarization [94]. Treatment of Fbn1 mgR/mgR mice with a monoclonal antibody directed against XGXXPG prevented elastin degradation, TGF-β signaling, upregulation of MMP-2 and −9, aortic macrophage infiltration, and suppressed the development of pulmonary emphysema [95]. Thus, inflammatory responses to EDPs are crucial in the progression of systemic pathology observed in Marfan syndrome patients. Marfan syndrome and ectopia lentis patient case study has shown increased ocular MMP-1, MMP-3, and MMP-9 as well as low TIMP-(1–3) levels implying that fibrillin mutations also increase degradation processes in the eye and result in ocular pathology [96,97]. While MMP-3 does not directly degrade elastin – it can activate pro-MMP-9 which likely contributes to degradation [98].

In Beals Hecht syndrome, fibrillin-2 mutation only effects the joints and tendons suggesting it is particularly important to the development and biomechanics of dense connective tissues. It was shown that fibrillin-2 containing structures are closely associated with flexor tendons and the pericellular matrix areas of tenocytes. Subsequently, experiments with fibrillin-2 null mice demonstrated that collagen content is unaffected but total crosslinking is reduced. Interestingly, while Beals Hecht syndrome is associated with extended limbs, fibrillin-2 null mice had focal areas of decreased bone length in the extremities [99].

Pathogenic mutations of fibrillin-1 and 2 often occur in the calcium-binding epidermal growth factor-like domains, it has been shown that calcium binding is required for secondary structure formation and lack of calcium stabilization increases susceptibility to proteolysis [100,101]. Given the importance of fibrillin in providing most of the microfibril scaffold composition for mature elastic fiber synthesis, it is unsurprising that mutations affecting this protein would also affect stability and cause disease in tissues where elastin is structurally important.

In the case of geleophysic dysplasia 2 and Weill-Marchesani syndrome type 2, these fibrillin-1 mutations effect binding regions of LTBPs/TGF-β and share very similar clinical presentations of disease. Fibroblasts isolated from a patient with fibrillin-1 TGF-β binding domain mutations that only displayed the joint and skin stiffening phenotype had overactivated TGF-β signaling, implying these mutations may predispose TGF-β to release from the elastic matrix. Dysregulation of TGF-β signaling was accompanied by upregulation of collagen and LTBP expression providing another mechanism linking clinical phenotypes to molecular alterations [102]. Increased collagen to elastin ratio would alter tissue elasticity potentially explaining the stiffness observed in skin and joints. Furthermore, increased expression of mutant LTBP like that in Weill-Marchesani syndrome type 2 could result in positive feedback initiating more TGF-β release. It has been shown that release of TGF-β from ECM can be mediated by fibrillin-1 fragments containing the TGF-β binding sequence. These fragments bind the N-terminal region of previously deposited fibrillin-1 in the insoluble cell layer, directly inhibiting the association of C-terminal LTBP-1 with fibrillin-1, subsequently releasing LTBP/TGF-β from the ECM [103]. Thus, fibrillin-1 fragmentation in Marfan syndrome or alteration of the TGF-β binding domain in other fibrilinopathies can further exacerbate TGF-β signaling and create positive-feedback loops.

### 3.3. Elastic fiber stability related pathological mutations

Mutations in genes for proteins involved with microfibrillar or elastin network integrity and organization cause similar hereditary diseases to elastinopathies and fibrillinopathies [53,54]. Mutant fibulin-4 creates unstable bridging between elastic fibers predisposing them to degradation. Accordingly, mutant fibulin 4 knock-in mice have increased MMP activity localized to the aorta as well as the lungs. [104]. Similar to fibrillinopathies, Weill-Marchesani syndrome type 3 and geleophysic dysplasia 3 mutations disrupt the calcium-binding epidermal growth factor-like domains of LTBP-2/3 while LTBP-4 mutations in autosomal recessive cutis laxa type 1C affect the fibulin 4/5 binding domains, which are the mutated proteins involved in autosomal recessive cutis laxa type 1A and B. Unsurprisingly given the nature of these mutations, Weill-Marchesani syndrome type 3 and geleophysic dysplasia 3 share more elastic fiber related pathological characteristics including heart valve defects and stiff skin while autosomal recessive cutis laxa type 1A-C causes vascular defects and lax skin. Given that LTBPs play similar structural roles in elastic fibers but disruption of each subtype results in distinctly different disease presentations, it is likely that they possess regional expression differences which predispose to varying conditions.

Mutations in the LOX gene lead to an insufficient crosslinking of elastin, causing AAs and aortic dissection as well as keratoconus, a condition in which the cornea assumes a conical shape and thins resulting in astigmatism eventually leading to vision loss [105–107]. ATP7A mutations in Menkes disease and occipital horn syndrome indirectly affect the crosslinking of elastin by disrupting cellular copper transport and subsequent LOX activity. These diseases have vastly different clinical phenotypes; however, share some common pathological features, such as loose skin and joints as well as occasional incidence of hernias [69]. Since dermal and connective tissue are affected in both these diseases, it could be inferred that LOX activity is required to either establish or maintain the mechanical properties of these tissues. Severe vascular phenotypes are more common in Menkes disease but, infrequently, arterial disturbances are also observed in occipital horn syndrome which could be explained by the level of disruption to ATP7A activity [108]. Menkes disease mutations result in total loss of function while occipital horn syndrome mutations are hypomorphic in nature. Pharmacological inhibition of LOX in mice *via* oral administration of β-aminopropionitrile results in spontaneous aortic dissection and when combined with angiotensin-II infusion causes aortic rupture implying LOX activity is also required for continued integrity of established elastic networks in the aorta [109]. Thus, mutations that effect LOX activity directly or indirectly induce elastic fiber related disease which varies in severity and tissue distribution.

Disruption of elastase inhibitors like alpha-anti trypsin 1 and TIMP-3 also causes pathogenesis likely in a similar manner to stimuli which increase expression or activity of MMPs. Alpha anti trypsin 1 deficient patients have larger abdominal aortas than healthy controls with increased stiffness and abnormal strain at a younger age [48,49]. Alpha anti trypsin 1 deficiency is also associated with development of severe emphysema, lung volume loss correlated to serum desmosine/isodesomine content demonstrating elastin degradation is directly linked to disease progression [110]. Sorsby fundus dystrophy is associated with ocular pathology but does not display arterial or pulmonary modifications observed in alpha anti trypsin 1 and LOX deficiency [75]. Mutant TIMP-3 expressing mice had symptoms of Sorsby Fundus Dystrophy with reduced MMP inhibitory capability and increased MMP-2 activity showing dysregulation of elastin degrading proteases was responsible for disease pathology [111]. In these diseases, indirect disruption of elastin matrix structure by modifying protease homeostasis results in systemic pathology with similar presentations to mutations of tropoelastin or fibrillin-1. It is unclear why some anti-protease mutations cause pathology in certain organs but have minimal effect to others despite the relatively widespread distribution of elastic fibers, however, it may result from tissue specific expression of proteases and their respective anti-proteases.

### 3.4. TGF-β related pathological mutations

TGF-β and LTBPs are closely involved in the formation of elastic fiber networks and TGF-β signaling is a direct regulator of ECM homeostasis [10]. Mutations in TGF-β pathway associated proteins also cause elastin degradation related diseases, paradoxically, these mutations can either suppress or enhance TGF-β signaling illustrating the dual role of this unusual cytokine. Shprintzen–Goldberg syndrome is a connective tissue disorder similar to Marfan and Loeys-Dietz Syndrome, however, while the former diseases are associated with excessive TGF-β signaling, Shprintzen–Goldberg syndrome patients have significantly attenuated signaling and less severe cardiovascular pathology. Mutated SKI, a transcriptional co-repressor of TGF-β signaling is not degraded after TGF-β receptor stimulation resulting in TGF-β signaling suppression [74]. Loss of function in the glucose transporter GLUT10 reduces glucose-stimulated expression of decorin, a TGF-β pathway inhibitor, and causes arterial tortuosity syndrome [50]. Arterial tortuosity syndrome shares many characteristics with Shprintzen–Goldberg syndrome however the former mutation reduces inhibition of TGF-β signaling while the latter increases inhibition of TGF-β signaling.

The mechanisms behind development of aortic pathology in TGF-β related mutations are not fully understood, however, evidence suggests that VSMCs and myeloid cells such as macrophages are intimately involved. SMAD4 variants reduce TGF-β transcriptional activity and are also associated with the development of thoracic AA by increased VSMC mediated proteolysis [112]. Subsequently it was shown that SMAD4 or TGF-β receptor II knockout in mouse VSMCs mediated development of AA and aortic rupture through upregulation of cathepsin S and MMP-12 leading to elastin degradation [113]. Interestingly, ablation of TGF-β receptor type II in myeloid cells of the mouse models of Marfan syndrome and monocyte depletion in SMAD4/TGF-β receptor type II knockout mice attenuate AA formation with reduced number of infiltrating macrophages [113,114]. Overall, data suggest the deleterious effect of mutations in the genes encoding proteins of the TGF-β pathway are mediated by effects on VSMC differentiation, proteolysis, and inflammatory cell crosstalk. However, further research is required due to conflicting effects.

### 3.5. Pyrophosphate homeostasis related pathological mutations

Disruption of pyrophosphate homeostasis proteins causes precipitation of calcium phosphate crystals on elastic fibers associated with their degradation in various tissues. Mutations in these proteins result in pseudoxanthoma elasticum, generalized arterial calcification of infancy (GACI), calcification of joints and arteries (CALJA), and arterial calcifications due to deficiency in CD73 (ACDC). The ABCC6 adenosine triphosphate (ATP)-dependent transmembrane transporter is responsible for extracellular nucleotide export, particularly ATP, which is converted into pyrophosphate and adenosine monophosphate by (E) NPP1. Adenosine monophosphate is subsequently degraded to inorganic phosphate (Pi) and adenosine by CD73 [115]. It has been shown pseudoxanthoma elasticum patients had higher desmosine serum content than controls associated with increased ankle brachial index but not correlated to calcification score, indicating development of peripheral artery disease in these patients is linked to elastin degradation [116]. These patients also show elevated systemic MMP-2 and 9, with dermal fibroblasts being a primary source of MMP-2 in the skin [117,118]. As discussed in the following section covering elastin degradation in cardiovascular and metabolic diseases, protease activity which degrades elastic fibers can be a critical step preceding the process of vascular calcification. Reduction of pyrophosphate in these diseases likely mediates increased activity of MMP-2 and 9 by their interaction with calcium phosphate and calcium pyrophosphate complexes. Calcium phosphate was shown to bind and aggregate MMPs while calcium pyrophosphate showed no activity, suggesting inorganic phosphate and pyrophosphate balance has a role in MMP localization [119].

### 3.6. Other pathological mutations involving elastin degradation

Mutations in β-galactosidase (*GLB1*), *N*-acetylgalactosamine-6-sulfate sulfatase (*GALNS*) and α-L-iduronidase (*IDUA*) genes lead to glycosaminoglycan accumulation on elastic fibers. It has been speculated that aortic dilation in these diseases is due to glycosaminoglycan mediated osmotic swelling causing separation of elastic lamella and increasing susceptibility to degradation [120]. In Hurler disease mice, upregulation of cathepsin S and MMP-12 was responsible for the degradation of elastin and aortic dilation [121]. Interestingly in Hurler disease patients, heparan sulfate accumulation on elastic fibers also prevented degradation mediated by cathepsin V showing differential regulation of elastin degrading proteases [122]. Supporting the hypothesis that glycosaminoglycans differentially regulate degradation, keratin sulfate inhibited MMP-2 in corneal and skin explant cultures but increased MMP-9 in skin explants. Other glycosaminoglycans such as dermatan sulfate and heparan sulfate also increased MMP-9 in skin explants [123]. Overall, despite pathogenesis being highly variable, modulation of water content and elastic fiber structure by glycosaminoglycans seemingly predisposes it to degradation in these diseases.

Disruptions of ADAM metallopeptidase with thrombospondin (ADAMTS) and ADAMTS-like (ADAMTSL) proteins also cause elastin-related hereditary disease due to their interactions with fibrillin-1 and LTBPs, however, their role is not clear. ADAMTS proteins possess proteolytic capabilities while ADAMTSL proteins are purely structural. ADAMTS10 and ADAMTS17 act as chaperones for fibrillin-1 and collagen type I while ADAMTSL4 binds fibrillin-1, enhancing its assembly [80,124,125]. ADAMTSL2 interacts with fibrillin-1 and may be involved in TGF-β signaling as geleophysic dysplasia type 1 patient’s fibroblasts secreted more latent and active TGF-β [60]. It is unclear how mutations in ADAMTS/L proteins cause pathogenesis, however, mutations in ADAMTS10 and 17 disrupt the metalloprotease and thrombospondin regions while the ADAMTSL2 and 4 mutations effect TGF-β binding domains. It is likely that ADAMTS proteins in these diseases are important to structure and predispose elastic fiber networks to degradation while ADAMTSL proteins also regulate TGF-β signaling by interacting with LTBPs.

Mutations in proline biosynthesis enzymes, delta-1-pyrroline-5-carboxylate synthetase (*ALDH18A1*), and pyrroline-5-carboxylate reductase 1 (*PYCR1*), causes reduced and smaller elastic fibers [57,126]. Assembly of tropoelastin and microfibrillar scaffolding into mature elastic fibers avoiding amyloid-like aggregation is intrinsically tied to the entropy of protein folding. Computer simulations of elastin-like peptide folding in aqueous medium demonstrated that assembly of elastomeric structures is favored over amyloid aggregation only when a certain threshold of proline/glycine composition is exceeded [127]. Proline and glycine residues increase disorder and hydration of peptides, hindering formation of larger aggregates by steric hinderance and entropic unfavourability respectively. Thus, disruption of proline biosynthesis may indirectly impact elastic fiber assembly resulting in the development of elastin degradation related pathology in these diseases. As was noted in Williams Beuren syndrome, hydroxyproline content seemingly only affected elastic fiber stability in the skin and not the aorta. Given that proline biosynthesis mutations are also associated with dermal but not cardiovascular manifestations, this strongly suggests that proline has a critical role in elastic fiber structure and stability within the skin.

*ATP6VOA2* proton pump mutations in autosomal recessive cutis laxa type 2A (ARCL2A) cause increased cellular apoptosis and elastin networks are fragmented into clumps [56]. Unique among molecular mechanisms previously discussed, *ATP6VOA2* mutations do not seemingly directly/indirectly affect structural components of the elastin network or TGF-β signaling but rather impact tropoelastin trafficking in lysosomes and the Golgi apparatus resulting in improper elastin networks due to reduced secretion. In this case, manifestations of disease are likely due to increased susceptibility of degradation in a similar manner to other elastinopathies, however, research is scarce due to the rarity of the disease.

Since Ehlers Danlos syndrome mutations primarily effect collagens and are not directly linked to elastin degradation, they are not discussed at length in this review. However, it is worth noting that Ehlers Danlos syndrome complications share substantial clinical overlap with systemic hereditary elastic fiber related diseases such as Marfan syndrome and Loeys Dietz syndrome. Ehlers Danlos syndrome manifestations vary greatly, however, incidence of cardiac and vascular abnormalities, hernias, as well as hyperextensible skin and joints have been observed [128]. Ehlers Danlos patients with the particularly deleterious vascular phenotype (type IV) have increased circulating serum TGF-β 1/2 and secretion of TGF-β from dermal fibroblasts suggesting dysregulation of TGF-β signaling is also implicated in pathogenesis [129]. Thus, although Ehlers Danlos syndrome is a collagen-related disorder, the appearance of similar clinical phenotypes to elastic fiber related hereditary diseases implies that the balance of collagen to elastic fiber is critical to ECM homeostasis and biomechanics as well as intrinsically tied to TGF-β regulation in many tissues.

## 4. Elastic fiber degradation in cardiovascular and metabolic diseases

Elastic fibers compose ~50 % of the arterial ECM, providing load-bearing ability in low-pressure regions and elasticity at higher loads for proper cardiovascular function in vertebrates [130]. Elastin is essential to maintain the “Windkessel Effect,” allowing sufficient blood supply to distal microvasculature [131]. The amount and organization of the elastic fibers dominate the passive mechanical behavior of the large arteries. Elastic fiber degradation is implicated in many cardiovascular diseases as well as metabolic deficiencies involving the vasculature. Arteries treated with elastase have a significant increase of lumen dimensions, indicating structural weakening [132–134]. Thus, insufficient elastic fiber, improper assembly, disorganization, or fragmentation of elastic fiber changes arterial biomechanics resulting in a malfunctioning circulatory system.

Vascular ECM remodeling after elastic fiber damage is characterized by the deposition of non-organized collagen fibers and proteoglycans, combined with changes to VSMC plasticity that stiffens the arterial wall leading to hypertension [135]. In AAs, elastin degradation in the aorta is directly associated with disease progression, including aortic dilation and rupture. Subsequently, several animal models of AA rely on direct application or injection of elastase to the aorta demonstrating that destruction of elastin is an initiating factor for this disease [132,136]. Increased expression of elastin degrading proteases such as MMP-2 and 9 and reduced expression of protease inhibitors such as cystatin c are also noted to occur in relation to AAs and atherosclerosis formation [137–139]. Elastin damage allows lipids and immune cells to enter and accumulate in the arterial wall leading to plaque formation and rupture during atherosclerotic lesion progression [34]. Release of LTBPs also modulates TGF-β activity, which increases migration and proliferation of the VSMCs in atherosclerotic plaque intima where they secrete matrix proteins that form the fibrous cap and neointima [141,121].

Elastic fibers have high-affinity sites for calcium ions which may be exposed after degradation [142,143]. Proteolysis of vascular elastin by cathepsins K, S, and V caused direct mineralization; X-ray spectroscopy of insoluble elastin predigested by cathepsins showed an 8-fold increase in the elemental percentage of calcium and phosphate compared to healthy elastin [11]. Even in the absence of proteolysis, hydrolyzed elastic fibers are prone to mineral deposition, as numerous charged sites promote adsorption of Ca^2+^ subsequently attracting phosphate and formation of hydroxyapatite crystals at nucleation sites [144]. Elastin calcification *in vivo* is associated with increased MMP expression and colocalization of MMP-2 and −9 to calcifying elastin fragments occurs in purified elastin subcutaneously implanted into rats [145–147]. Peri-adventitial application of calcium chloride to the abdominal aorta causes inflammation and dilation along with destruction and calcification of elastic lamella. However, aortas from both MMP-2 and −9 knockout mice do not calcify, suggesting MMP-mediated elastin degradation is a prerequisite for calcium chloride induced vascular calcification *in vivo* [148].

Deposition of calcium phosphate on degraded elastic fibers in the medial layer of the arteries, known as medial arterial calcification, contributes to increased arterial stiffness and high cardiovascular co-morbidity in chronic kidney disease (CKD) patients. Arterial stiffness in rodent models of CKD is linked to VSMC osteogenesis and degradation of elastin lamellae [149,150]. Subsequently, CKD patients have increased serum levels of MMP-2, cathepsin S, and EDPs, suggesting that elastin degradation is linked to vascular calcification processes in this disease [151,152]. Serum phosphate is correlated to MMP-2 expression and high levels of inorganic phosphate stimulate secretion of MMP-2 and 9 from rat VSMCs, linking elevated phosphate due to kidney dysfunction to elastin degrading protease expression [153,154]. As pathology progresses to end stage renal disease, elastic fiber modification increases. Patients with end stage renal disease have more pentosidine and malondialdehyde adducts than those with early disease suggesting that elastin is modified by both glycoxidation as well as lipid peroxidation as the disease progresses [155]. Unfortunately, treatment of CKD and end stage renal disease *via* hemodialysis results in even greater pentosidine and calcium deposits on medial aortic elastin which increase over time [156]. Thus, the prognosis for those with end stage renal disease undergoing dialysis is typically poor, as although dialysis treatment may temporarily remove toxic products from the blood, it accelerates the process of vascular calcification further reducing kidney function.

Elastic fiber loss, oxidative stress, and neovascularization occurring in diabetic patients lead to development of a chronic inflammatory response and complications, including angiopathy. Subsequently, increased arterial stiffness linked to a decreased elastin to collagen ratio is seen in diabetic humans and mice [157,158]. Elastic fiber degradation is also implicated in hepatic disease; elevated serum levels of EDPs, and urine concentration of desmosine and isodesomsine were seen in cirrhosis patients [159,160]. In obese people, non-alcoholic fatty liver disease markers were positively correlated with serum EDPs and further increased in those with diabetes [22]. There is a strong association between expression of neutrophil elastase and suppression of its inhibitor alpha 1-antitrypsin and occurrence of non-alcoholic fatty liver disease [161]. Knockout of neutrophil elastase showed a protective effect against western diet induced nonalcoholic steatohepatitis by influencing ceramide metabolism [162]. Given that neutrophil elastase activity can generate EDPS which activate Neu-1 and its subsequent activity promoting conversion of gangliosides to ceramides, it is likely that elastin degradation is a root cause of this disease.

Elastic fiber is also a significant component of the heart tissue and cardiac structural abnormalities are observed in various elastin related hereditary diseases. Mechanical testing of elastic fibers isolated from porcine aortic valves suggest elastin is required to return collagen fibers to a resting state after loading cycle completion [163]. Elastase digested porcine aortic valves show significant increase in mean axial curvature when subjected to cyclic flexure, demonstrating that elastic fibers are critical to heart valve function [164]. Juvenile Eln +/− show normal valve function however, incidence of aortic valve dysfunction such as regurgitation progressively increases with age [165]. This evidence suggest that very little elastin is required to maintain the function of the aortic valve, however, disruption of elastin by hereditary mutation or progressive degradation with age and mechanical strain causes disease.

As post-myocardial infarction (MI) cardiac remodeling progresses, elastin is replaced by fibrous collagenous tissue, and EDPs can be detected in patient serum [166,167]. Plasma desmosine content is directly correlated to adverse outcomes post-MI suggesting that continued elastin degradation during cardiac remodeling exacerbates disease pathogenesis [168]. Cardiac remodeling in type 2 diabetes mellitus is also linked to elastin degradation and autoimmunity to elastin, anti-elastin antibodies were significantly elevated in type 2 diabetes patients and linked with progression of left ventricular hypertrophy [169]. Unsurprisingly, left ventricular hypertrophy and remodeling is associated with increased expression of various elastolytic proteases such as MMP-2 and MMP-9 [170,171]. Interestingly however, MMP-2 knockout exacerbates ventricular hypertrophy in angiotensin II infused mice suggesting a dual role for this protease in regulating cardiac tissues [172].

It was recently shown that Purkinje cells are closely associated with highly organized elastic fiber networks which, in coordination with collagen, provide mechanical support during systole and diastole respectively [173]. Given the role of Purkinje cells in cardiac electrophysiology as well as the ability of EDPs to induce Ca^2+^ signaling, there may be some association between destruction of these specific structures and electrical disturbances. Supporting this hypothesis, specific inhibition of MMP-9 prevented angiotensin II induced ventricular arrythmia in mice by modifying calcium homeostasis [174]. Although not typically a primary manifestation of disease, cardiac arrhythmia incidence is increased in populations with heritable thoracic aortic disease caused by fibrillin-1 or TGF-β pathway mutations suggesting there may be a correlation between elastin degradation and cardiac electrophysiology. These arrythmias were noted to occur in younger patients who had yet to develop significant valvular or aortic disease, implying that the molecular root cause was independent of cardiovascular comorbidities [175].

Cardiovascular and metabolic disease is closely associated with degradation of elastin. Diseases primarily caused by metabolic disruption such as diabetes and non-alcoholic fatty liver disease are intrinsically tied to cardiovascular disease due to the inflammatory feedback associated with systemic modification and degradation of vascular elastic fibers. Those with diabetes are predisposed to develop atherosclerosis, cardiovascular calcification, and MI [176,177], while those with non-alcoholic fatty liver disease are predisposed to CKD [178]. Similarly, existing cardiovascular disease can induce or exacerbate the pathogenesis of another cardiovascular disease, as there is a close association between coincidence of atherosclerosis, AA, and heart disease [179]. Overall, elastin acts as a common link in dysregulation between the metabolic and cardiovascular systems associated with clinical manifestations of disease. Further research regarding the effect of disruption of elastic fiber or ECM networks on cardiac disease, especially electrophysiological disturbances, is warranted.

## 5. Elastic fiber degradation in pulmonary diseases

In the lung parenchyma, the elastic fibers establish lamellar sheets surrounding the alveoli, making up 20–30 % of the dry tissue weight [180]. The elastin present in the lung alveoli allows balloon-like stretching of lungs during inhalation and restoration of lung volume during exhalation due to elastic recoil. Elastin is vital to the lungs’ function, stretching to 140 % of its resting length, whereas collagen can stretch only 2 % [180,181]. In rat models of lung development, copper deficiency or β-aminopropionitrile mediated LOX inhibition impairs alveolar septal formation suggesting elastic fiber crosslinking is critical to generate these tissue structures [182,183].

Elastin and fibrillin gene mutations highlight the importance of elastin to lung development and suggest that loss of elastin is linked with susceptibility to destructive lung disease. *Eln* −/− mice die within 48 h of birth due to abnormal lung growth, and *Eln* +/− mice exposed to cigarette smoke develop more severe airspace enlargement than wild-type mice [180]. Similarly, mice with 30 % of wild-type tropoelastin expression have enlarged thoracic cavities and larger lungs than both wild-type and *Eln*+/− lungs at the same pressure [180]. Mutations in elastin-related genes such as fibulin-4 or 5, fibrillin-1, LTBP, and LOX-like 1, cause lung disease in humans and mice. Consequently, alpha anti trypsin 1 deficiency and autosomal dominant cutis laxa mutations have also been associated with severe emphysema or chronic obstructive pulmonary disease.

Agents that cause pulmonary damage and fibrosis include oxidizers, oxides of sulfur and nitrogen, cigarette smoke, inorganic dust, or pollution, and drugs or chemicals such as bleomycin. Lung damage involves degradation of elastic fiber, followed by fibrosis of the tissues that impact elasticity and breathing capacity. Scar tissue is characterized by parenchymal destruction and pathologic fibrosis, as seen in idiopathic pulmonary fibrosis. The fragmentation and replacement of elastic tissue with dense collagenous tissue and decreased ECM turnover is a hallmark feature of the disease. Desmosine and isodesmosine fragments are detectable in the plasma or urine of smokers as well as chronic obstructive pulmonary disease, cystic fibrosis, emphysema, and idiopathic pulmonary fibrosis patients, correlating with disease progression [184–188]. Intrapulmonary instillation of elastase and inhaled aerosolized EDPs produce lesions like those in clinical emphysema. Thus, these stimuli are frequently used for animal models of pulmonary disease related to elastin degradation and fibrosis [189]. Unsurprisingly, expression of elastin degrading MMP-12 was inversely correlated with the carbon monoxide diffusing capacity of the lung and alveolar volume in patients with emphysema [190]. Consequently, MMP-12 and neutrophil elastase −/− mice were drastically protected from cigarette smoke induced emphysema due to reduced propensity for elastin degradation [191,192].

In a similar manner to the coincidence of metabolic and cardiovascular diseases, pulmonary disease increases the propensity for cardiovascular co-morbidities. Bronchiectasis, COPD, and emphysema are associated with increased cardiovascular disease risk [193]. In COPD patients, serum MMP-2 and neutrophil elastase are increased suggesting that coincidence of cardiovascular disease may be due to systemic elastolytic protease activity [194]. Supporting this hypothesis, serum desmosine levels in bronchiectasis patients was directly correlated to increased all-cause and cardiovascular related mortality [195]. Thus, systemic inflammation stemming from elastin degradation in pulmonary disease may perpetuate disease and promote pathogenesis in other organs.

Overall elastin is critical to lung structure and function, furthermore, its degradation produces inflammation and pulmonary pathology associated with auto-immune responses to degraded elastin. Stabilizing elastin and preventing the release of EDPs may help reduce the pathogenesis of pulmonary disease. However, given that the continuum of lung disease extends from excessive matrix deposition and fibrosis as observed in idiopathic pulmonary fibrosis to complete destruction of pulmonary tissue structure as seen in severe emphysema, more thorough research is needed to understand the effect of ECM turnover and balance.

## 6. Elastic fiber degradation effects on cancer

Most research related to elastic fiber degradation and cancer is focused on the effects of EDPs that can enhance cancer pathogenesis, such as proliferation, suppression of apoptosis, chemotaxis, angiogenesis, and protease secretion. EDPs cause endothelial cell proliferation, migration, and tubulogenesis, an essential factor in cancer metastasis [196]. Both mature elastic fibers and soluble κ-elastin adhere to highly metastatic human lung carcinoma (3LL-HM) and melanoma cell lines (A-2058); while less invasive Lewis lung carcinoma and rhabdomyosarcoma cells have lower affinity [197]. Elastin binding may increase the invasiveness of cancerous cells by providing a mechanism for vascular adherence and angiotropism. Presence of extravascular tumor cell abluminal angiotropism is observed in melanomas, prostate cancer, as well as lymphomas. In cases of uveal melanoma, angiotropism of tumorous cells was directly associated with metastasis and patient mortality [198–200]. In fibrosarcoma, glioblastoma, astrocytoma, melanoma, and lung cancer cells: EDPs enhance MMP expression, chemotaxis, and proliferation, promoting metastasis [201–203]. Consequently, subcutaneous EDP injection increases melanoma size [204]. Elastin and MMP-9 expression are upregulated in colorectal cancer tumor tissues and cancerous epithelial cells as opposed to healthy tissues and cells from the same donor. Furthermore, EDPs increased the proliferation of colon cancer epithelial cells [32]. Overall, the inflammatory environment associated with cancer development and generation of EDPs contributes to pathological feedback loops that advance the progression and severity of cancer.

Preventing the degradation of elastin might therefore block positive inflammatory tumor feedback and may result in reduced cancer progression and metastasis. Furthermore, the ability of EDPs to induce migration and angiogenic effects in endothelial cells may participate in carcinogenesis and warrants continued investigation. While elastin is important to tissue architecture and possesses unique mechanical properties which have been studied more extensively in cardiovascular and pulmonary disease, little work has been done related to biomechanics of elastin and carcinogenesis. It has been shown tissue stiffness can also influence cellular processes conducive to carcinogenesis such as migration, induction of epithelial/endothelial to mesenchymal transition, as well as angiogenesis [205–207]. Thus, future research into the influence of elastin on cancer pathogenesis should include evaluations of matrix composition and relation to biomechanics.

## 7. Elastic fiber degradation in infectious diseases

The role of elastic fiber degradation in infectious disease pathogenesis is not entirely understood; however, it may influence microorganism attachment and invasion and alter local immune responses.

### 7.1. Viruses

The global response to SARS-CoV-2 increased focus on understanding the pathogenesis of respiratory viral infections. Viral infections are linked to lung fibrosis as well as the development of obstructive pulmonary diseases. Similarly, the presence of pre-existing pulmonary conditions predisposes individuals to infection. Infants are more susceptible to viral infections due to extensive airway remodeling, with infections linked to the development of structural and functional lung deficits in asthmatic children. Viral inflammation can affect the ECM remodeling process surrounding elastin synthesis and alveoli development, suggesting a plausible mechanism [208].

The role of elastolytic enzymes in the development of irreversible lung pathology during SARS-CoV-2 infection and potential therapeutic use of protease inhibition was recently extensively reviewed by Boraldi et al. [209]. There is strong evidence linking SARS-CoV-2 related complications to protease/anti-protease balance and permanent destruction of elastic tissue. In SARS-CoV-2 infection, increased elastase expression with decreased anti-protease activity and elastin degradation are hallmarks of diseased lung tissues in critically ill patients [210,211]. Elastase has been shown to cleave SARS-coronavirus spike proteins resulting in activation, increased binding, and higher intracellular viral load [212,213]. SARS-CoV-2 variants which possessed an elastase cleavage site were more likely to spread in areas with higher prevalence of alpha 1 antitrypsin deficiency, relating anti-protease expression to disease susceptibility [214]. Interestingly, elastase inhibition improves the effectiveness of alum adjuvants in SARS-CoV-2 vaccines to promote resolving systemic and mucosal immunity indicating that elastin degradation may critically alter immune responses during infection [215]. The presence of degraded elastin may enhance basal elastase expression *via* inflammatory cell positive feedback, thereby increasing the infectivity of SARS-CoV-2. Alternatively, EDPs may exacerbate inflammatory polarization and autoimmunity, preventing natural progression to pro-resolving tissue repair cellular phenotypes.

Rhinovirus infection is the underlying cause of the common cold. Mice exposed to both elastase and lipopolysaccharide (LPS) were more susceptible to rhinovirus infection than either elastase or LPS alone with increased lung hyper-responsivity and size during infection. Furthermore, expression of inflammatory cytokines and viral RNA were observed up to two weeks post-infection. Antiviral cytokines such as interferons, interferon response factor-7, and IL-10 were also downregulated in response to LPS + elastase treatment [216]. The addition of elastase to LPS may elicit a more significant inflammatory response and promote viral infection due to the release of EDPs. Patients with cystic fibrosis or on machine-assisted airway ventilation are often co-infected with *Pseudomonas aeruginosa* bacteria and influenza A virus. It was shown *in vitro* in several human bronchial epithelial cell lines and *in vivo* using C57Bl/6 mice that MMP inhibitors abrogated influenza A induced increase in MMP-9 expression and subsequent secondary *P. aeruginosa* infection. Influenza A infection post-transcriptionally inhibits the anti-elastase protein known as elafin/trappin-2, which protects against destructive inflammatory neutrophil influx [217]. These data suggest preservation of elastin may be beneficial in preventing viral/bacterial co-infection. Viral infections also have been linked to elastin degradation and fibrosis of the liver. Desmosine and isodesmosine detected in the serum and urine of patients with cirrhosis correlates with hepatic fibrosis score in liver disease caused by hepatitis C or alcohol consumption [159]. Viral infection may trigger inflammatory responses that include upregulation of elastase. The subsequent disruption of ECM turnover balance could result in development of fibrosis, as collagen is deposited to replace elastin.

### 7.2. Bacteria

Like viral infections, bacteria that infect the lungs are closely associated with the degradation of elastic fibers. *Mycobacterium tuberculosis* is a gram-positive facultative intracellular bacterium that causes tuberculosis, a highly contagious and damaging lung disease. The antigen 85 complex is a surface bound virulence factor required for intracellular *M. tuberculosis* survival within macrophages which has been shown to prevent formation of phagolysosomes. The antigen 85 complex also facilitates improved adherence, invasion, and dissemination [218]. Elastin and tropoelastin derived from human aorta, lung, and skin were shown to bind to antigen 85 demonstrating elastin degradation and synthesis may be chemotactic to *M. tuberculosis*. Subsequently, Caco-2 cells transfected with elastin targeting siRNA had extracellular binding of antigen 85 reduced by approximately 34 %, suggesting the presence of elastin is crucial for adherence and subsequent intracellular invasion of *M. tuberculosis* [218]. Macrophages incubated with *M. tuberculosis* dose-dependently increase secretion of MMP-2 and 9, demonstrating that inflammatory responses to bacterial infection can also induce elastin degradation and potentially participate in inflammatory feedback loops promoting bacterial adhesion to elastin *via* the antigen 85 complex [219]. MMP expression and the importance of antigen 85 elastin binding implies that elastin plays an essential role in susceptibility to mycobacterial infection. Isodesmosine was chemotactic to 12 of 15 different isolated *P. aeruginosa* clinical specimens suggesting that damage to elastin may promote bacterial colonization [220]. Elastase produced by *P. aeruginosa* can degrade pulmonary surfactant proteins A and D which possess anti-microbial properties and positively modulate host immune responses to resolve infection [221,222]. Two epitopes of *P. aeruginosa* elastase can induce production of proteolytic activity inhibiting antibodies *in vivo*. Subsequent immunization of rats with these peptides conjugated to either keyhole limpet hemocyanin or tetanus toxoid decreased *P. aeruginosa* and *Burkholderia cepacia* infection associated with reduction of leukocyte infiltration, bronchoalveolar lavage fluid, and pathological tissue remodeling [223].

Elastin degradation may also be involved in other bacterial infections unrelated to pulmonary pathology. Pathogenic *Leptospira sp.* are Gram-negative spirochete bacteria that can enter a host through the eyes, mouth, nose, or cuts. A crucial step to Leptospira pathogenesis and chronic infection is host cell surface and ECM adhesion; consequently, its OmpL37 surface protein dose-dependently binds to human dermal elastin until saturation [224]. Leptospira binding to dermal elastin may represent a key first step in establishment of infection, as exposed dermal elastin would be present within open wounds.

### 7.3. Fungi

In a similar manner to viral and bacterial pulmonary pathogens, fungal colonization is associated with lung elastin degradation. *Aspergillus fumigatus* is an opportunistic pathogenic fungus known for angiotropism and accounts for 90 % of cases of pulmonary aspergillosis in immunosuppressed patients. *A. fumigatus* elastase activity is considered a vital pathogenicity factor, in experiments with immunocompromised mice, only *A. fumigatus* strains that expressed elastase caused infection and subsequent mortality [225,226]. *In vitro*, 10 mg/mL of soluble elastin significantly increased biofilm development, stimulating the production of ECM components in *A. fumigatus* cultures suggesting the presence of elastin promotes colonization. However seemingly paradoxically, mycelium surface hydrophobicity, a measure of the ability of mycelia to hydrophobically bind surfaces was also decreased [227]. In this context, the presence of elastin and reduced hydrophobicity may enhance the dissemination of *A. fumigatus* by promoting movement/detachment of established fungi. Co-culture experiments on unmodified and Congo red containing agar showed that *P. aeruginosa* cultured with mature *A. fumigatus* biofilms produced significantly more elastase in 60 % of tested strain isolates and supernatants from co-culture were more cytotoxic to human lung epithelial cells than *P. aeruginosa* supernatant alone. Interestingly, the secretion of elastase from *P. aeruginosa* had an inhibitory effect on the growth of *A. fumigatus* colonies when both pathogens were cultured in proximity on normal agar [228]. These data demonstrate that the frequent clinical co-occurrence and symbiotic relationship between *P. aeruginosa* and *A. fumigatus* may depend on modification of the host elastic network in the lung, as these pathogens display competitive behavior when isolated *in vitro*. The synergistic enhancement of elastase secretion in *P. aeruginosa* – *A. fumigatus* co-culture may create a more permissive environment for both infectious species by destroying the elastic fiber/epithelial cell barrier of the lung parenchyma and degrading surfactant proteins.

### 7.4. Parasites

Schistosomiasis is a parasitic infection of the urinary tract or intestines caused by flatworms known as schistosomes. Symptoms include abdominal pain, diarrhea, blood in stool or urine, and infection can lead to liver fibrosis. *Schistosoma mansoni* larvae were shown to degrade human dermal elastin, which in coordination with other proteases assists in facilitating entry through the dermis [229]. Mice infected with *S. mansoni* excrete excess pyridinoline crosslinks in urine correlated with collagen content of liver granulomas, suggesting a link between elastin degradation and liver fibrosis progression [230]. Like hepatic viral infections, schistosomes may express elastases to facilitate tissue invasion and colonization which impact ECM turnover balance and result in a fibrotic response. As observed with *Leptospira sp.*, elastase expression appears to promote infection by degrading dermal elastin, which may act as a barrier to microbial infiltration.

## 8. Elastic fiber degradation in dermal disease

Alterations to elastic fibers from genetic or acquired deficiencies cause the manifestation of cutaneous disease. For many previously described genetic diseases, the dermal manifestations could generally be described as varying forms of cutis laxa [231]. While several factors such as UV radiation, genetic disorders, and autoimmune or foreign body reactions are linked to the development of dermal diseases, pathogenesis is still poorly understood [231]. Dermal diseases related to elastin typically present as an alteration in skin elasticity or appearance of abscesses. In anetoderma, cutis laxa, elastoderma, and mid dermal elastolysis, the primary clinical observation is abnormally lax skin. Anetoderma cultured patient fibroblasts show upregulation of MMP-3, 7, and 9 as well as downregulation of TIMP-2 suggesting dermal pathology may be related to elastin-degrading protease balance [232]. Likewise, cultured cutis laxa patient fibroblasts express significantly more MMP-1, 3, and 9 with dermal lesions showing elevated MMP-3, −9, and −12 activity [233,234]. In cases of cutis laxa with no clear genetic cause, dermal fibroblast elastolytic activity was increased compared to healthy controls suggesting elastin degradation in a critical step in manifestation of these diseases regardless of etiology [235].

Like pathologies induced by LOX mutation or copper metabolism alterations, the copper-chelating agent D-penicillamine used to treat another disorder of copper metabolism known as Wilson’s disease is associated with acquired dermal elastic fiber diseases including elastosis perforans serpiginosa [236,237]. Perforating calcific elastosis is primarily observed in obese women and associated with incidence of renal failure, suggesting deposition of calcium phosphate precipitates on elastic fibers occurs in a similar manner as seen in CKD and in genetic diseases where extracellular pyrophosphate metabolism is altered [238]. In elastoma and myxedema, tissue mass formation is observed due to the deposition of adipose tissue and mucopolysaccharides in regions of dermal elastin degradation similar to Hurler Disease [231,239,240]. Alteration of fiber structure induces ECM remodeling and, consequently, pathology as the tissue architecture is degraded.

Elastolytic giant cell granuloma and granulomatous slack skin are characterized by lax skin and erythematous plaques in the dermis. These regions of skin display degraded elastin and heavy foreign body giant cell infiltration resulting in granuloma formation [231]. Elastolytic giant cell granuloma lesions have enhanced expression of MMP-12 localized to macrophages while progression of dermal T-cell malignancy (mycosis fungoides) into granulomatous slack skin is associated with increased expression of MMP-2 and 9 [241,242]. Consequently, it was shown that MMP-9 is required for macrophage fusion into foreign body giant cells and that giant cells secrete substantially more MMP-9 than mononuclear monocytes/macrophages [243,244]. Foreign body giant cell and granuloma formation typically occurs in response to large materials which cannot be phagocytosed by macrophages as particles exceed the volume of the cell [245]. Due to amphiphilic nature, EDPs can self-assemble into nano and micro-structured materials under *in vivo* conditions with some structures far exceeding the approximate 20 μM diameter of human macrophages [246]. Increased concentration of EDPs correlates with increasing self-assembled aggregate size suggesting that giant cell fusion and granuloma formation may also be linked to the degradation of elastin and presence of globular aggregation of EDPs. Thus, elastic fiber degradation may be pre-requisite to formation of granular lesions in the skin.

Overall, alterations to dermal elastin are associated with disease pathology and related to biomechanical properties as well as the potential of damaged elastin to induce inflammation or facilitate binding and deposition of abnormal material *in vivo*.

## 9. Elastic fiber degradation in ophthalmic disease

Elastic fibers are present in retinal vessels and comprises 3.73 ± 0.55 % of the tissue weight [247]. Elastin is an integral part of the Bruch’s membrane structure within the eye, and EDPs are associated with the development of ocular pathology. Elastin-related hereditary diseases such as ectopia lentis, Marfan syndrome, pseudoxanthoma elasticum, and Sorsby fundus dystrophy can cause visual impairment and ocular pathology illustrating that elastic tissues are critical to function [75,248]. In Marfan syndrome and LOX mutations, corneal elastin is altered with abnormal eye geometry, indicating a structural role for elastic fibers in optical tissues [105,249].

Mutations or overexpression of HTRA1, an elastase known to be a risk factor for age related macular degeneration, result in the destruction of elastin in Bruch’s membrane and development of choroidal neovascularization [250,251]. Age related macular degeneration, pseudoxanthoma elasticum, and Sorsby fundus dystrophy are characterized by the presence of choroidal neovascularization, consequently, EDPs induce choroidal endothelial cell migration implicating a role in this pathology [33]. Serum EDPs were increased in patients with age related macular degeneration relative to control subjects, and subjects with neovascularization had higher plasma concentrations of EDPs than those with early disease [252]. Overall, evidence suggests that degradation of Bruch’s membrane and release of EDPs are intrinsically tied to the process of neovascularization in the eye and correlated to vision loss.

Unique from other ophthalmic diseases discussed, pseudoexfoliation syndrome is a systemic disease of unknown origin characterized by fibril aggregates in the heart, kidneys, liver, lungs, and particularly the eyes. Fibrillin-1 is composed of ~13 % cysteine residues which can be modified to homocysteine, making it prone to aggregation, thereby contributing to formation of plaques and pathogenesis in pseudeoexfoliation syndrome [253,254]. Coincidently, Marfan syndrome patients with cysteine residue modifying mutations of the fibrillin-1 calcium binding epidermal growth factor like domain all showed sign of retinal detachment or ectopia lentis [255]. Diabetic retinopathy and rhegmatogenous retinal detachment are also associated with increased homocysteine levels suggesting that modification of elastic fibers by this amino acid is particularly detrimental to the structural integrity of the eye [256]. Beyond its modification of fibrillin-1 structure, homocysteine can increase expression of elastolytic proteases as well as suppress LOX mediated ECM crosslinking in diabetic retinopathy and rhegmatogenous retinal detachment [257,258]. Elevated homocysteine is correlated with cardiovascular, metabolic, as well as ophthalmic elastin related diseases suggesting this biomarker is closely associated to the degradation of elastic fiber and potentially participates in systemic co-morbidity [259,260]. Research surrounding elastin and its role in the pathogenesis of ophthalmic disease is minimal compared to more elastin rich tissues such as the cardiovascular system. Given that many genetic diseases that impact elastin also cause eye pathology, an increased focus should be placed on understanding the role of elastin to preserve eye function and structure.

## 10. Elastin degradation in dense connective tissue pathology

Elastin is present in dense connective tissues such as tendons, joints, and reproductive tissues. Defects in elastin synthesis or damage to elastin networks can disrupt the proper function of these tissues, causing pathological manifestations. In tendons and ligaments, elastin constitutes ~1–5 % of the dry tissue weight; however, it provides up to 70 % of load support during transverse tensile and shear deformation of the medial collateral ligament. Elastase treatment resulted in a 2–3 fold reduction of elastic modulus and 60–70 % reduced peak stress tolerance [261]. Similarly, elastic fibers are increased in higher load-bearing tendons and decrease with age, linking loss of elastin to the propensity for tendon injury or stiffness [262]. Articular cartilage is primarily composed of collagen; however, elastin and microfibrils are also present in all parts of the tissue. Elastic fibers in the superficial zone are aligned parallel to the articulating surface, extensive branching and prevalence of elastic fiber network in this region indicate its mechanical importance corresponding to the high shear stress of joint articulation [263]. As observed in elastin-related hereditary diseases, the effect on joint biomechanics can be highly variable, with both excessive stiffness and flexibility observed.

In herniated abdominal walls, elastin is increased, but fibers are thick, winding, and fragmented [264]. It is unclear how this alteration of elastic fibers results in herniation; however, structural disruption may result in abnormal response to mechanical stress with increased stiffness and reduced tear resistance. In direct inguinal hernia patients, a characteristic ECM alteration of the transversalis fascia is decreased density of oxytalan elastic fibers composed entirely of microfibrils lacking elastin. Since oxytalan fibers have been demonstrated to provide strength against tearing forces, it is likely that the increased expression of elastin and altered remaining mature fibers provide little mechanical resistance to tearing in the fascia [265]. Interestingly, there is also an association between the incidence of aortic aneurysmal disease and hernia development, which implies there is a systemic cause that could be linked to elastic fiber degradation [266,267]. Fibulin-3 knockout mice have reduced fascial elastin resulting in inguinal hernia and pelvic organ prolapse development [268,269]. Fibulin-3 minimally interacts directly with ECM components such as elastin or fibrillin-1 however it strongly regulates TIMP-3, indicating reduction of fibulin-3 may increase aberrant elastic fiber degradation through protease/anti-protease imbalance in a similar manner to Sorsby Fundus Dystrophy [269]. Pathogenic elastin remodeling can also occur in vaginal tissues post-childbirth and is linked to the development of pelvic organ prolapse. Patients with this disease have reduced elastin mRNA synthesis and fiber content in uterosacral ligaments as well as reduced fibulin-5 expression [270,271]. Female pelvic floor disorder patients also display evidence of elastin alterations, neutrophil elastase activity is increased while alpha anti trypsin 1 expression is decreased suggesting protease activity is a factor in pathogenesis [272].

In *Eln*+/− mice, penile elastin content was reduced by ~33 % and associated with increased VSMCs and contractility. These changes and impaired veno-occlusive function suggest that *Eln*+/− mice may exhibit erectile dysfunction [273]. In Peyronie’s disease patients, disruption of elastin or a decrease in elastin content has been reported and can result in penile deformities during erection and erectile dysfunction [274]. Like many other diseases involving elastin degradation, Peyronie’s disease is associated with increased serum levels of anti- tropo and alpha elastin antibodies suggesting an auto-immune component is involved in pathogenesis related to elastin synthesis (tropoelastin) as well as elastin destruction (alpha-elastin) [275]. Incidence of Peyronie’s disease is linked with occurrence of another connective tissue disorder known as Dupuytren’s contracture, where abnormal thickening of fascial tissue in the hand prevents extension of digits [276]. Elastase digestion of healthy hand fascial tissue caused a reduction of Young’s modulus and increased extensibility with linear strain positively correlated to total elastin content. This affect was not observed in contracture bands of Dupuytren’s patients implying that elastic fiber content and structure are critical to the elasticity and mobility of these tissues [277]. In Dupuytren’s contracture affected tissues, MMP-2, TGF-β1, and decorin are significantly upregulated implicating elastin degradation and TGF-β signaling dysregulation [278]. Given that these factors are also directly associated with systemic pathology in various hereditary diseases, co-incidence of Peyronie’s disease and Dupuytren’s contracture may be due to common molecular root causes. Indeed, it was recently shown that Peyronie’s disease-like symptoms could be induced in rabbits by subtunical injection of recombinant TGF-β. Erectile dysfunction was accompanied by elastin loss and upregulation of elastase 2B, interestingly however, expression of MMP-2 and 9 was decreased in lesions [279].

Between ophthalmic and connective tissue disease, floppy eyelid syndrome is a condition where patients cannot move their eyelid properly and it remains loose on the face. Elastase digestion of cadaver upper eyelid tarsus tissue showed reduction of Young’s modulus from control tissue mimicking the effect of floppy eyelid disease, suggesting elastic fiber integrity maintains eyelid retention [280]. Both MMP-7 and 9 are upregulated in floppy eyelid disease and histology reveals damaged elastic fibers that likely result in clinically observed mechanical dysfunction [281,282]. In dense connective tissue diseases related to elastic fiber degradation, some manifestations closely mirror processes observed during aging. Three dimensional imaging of elastic fiber networks in young and aged abdominal as well as eyelid skin showed drastic reductions in oxytalan elastic fibers and reduction of fibrillin-1 expression indicating that microfibrillar structures are vital to mechanics of these tissues [283]. Overall, elastic fiber has a crucial mechanical role, and defects can affect dense connective tissue function. Further research should have increased focus on if pathology primarily results from biologically or mechanically mediated changes within dense connective tissue.

## 11. Summary and discussion

Elastic fiber degradation is observed in many common disorders as summarized in Table 2. As seen in genetic diseases that impact elastic fibers, disruption causes systemic effects in many tissues with the root cause being degradation of elastin. Elastin damage reduces tissue elasticity and releases EDPs as well as TGF-β which induce various pathogenic effects. The imbalance towards inflammatory or de-differentiated cellular phenotypes causes further elastin degradation and results in disease pathogenesis. Preventing this feedback by preserving the non-regenerable elastin network could be vital to reversing pathogenesis. Diseases involving elastin degeneration have no pharmacological therapy, resorting to surgical intervention or treatment of symptoms. Thus, therapies related to elastic fiber stabilization may provide an ideal solution to address the root cause of these diseases.

Strategies to preserve elastic networks by preventing proteolytic degradation have shown some success in preventing elastin related diseases. MMP inhibition by doxycycline stopped Marfan syndrome aneurysm development in humans and animals, preventing bleomycin-induced transient fibrosis in mice [284–286]. The seemingly paradoxical decrease of lung fibrosis with inhibition of MMPs could be explained by regulation dependency among different MMPs – with selective inhibition leading to up-regulation of anti-fibrotic MMPs. In a similar manner, dual inhibition of MMP-9 and 12 was effective in reducing cigarette smoke-induced airspace enlargement by 70 % in guinea pigs [287]. Pre-treatment of purified bovine neck ligament elastin by immersion in a 0.1 M aluminum chloride (AlCl_3_) solution prevented degradation and calcification after subsequent sub-dermal implantation into rats for up to 30 days due to irreversible aluminum ion binding and alteration of elastic fiber structure [147,288,289]. In mouse models of MI, neutrophil elastase inhibition enhanced survival and cardiac function associated with a reduction of fibrosis as well as infiltration of macrophages [290]. Inhibiting the activity of elastin degrading enzymes can prevent pathological progression by preserving mature elastic fibers and preventing release of pro-inflammatory EDPs, however, MMP-inhibition can only limit, not repair, degraded elastin.

Several methods for restoring elastin networks have been explored including cell therapies, genetic engineering approaches, and directed cellular elastin synthesis. Implanting recombinant elastin-producing bone-marrow stromal or COS-7 cells in MI scars reduced expansion and prevented dilation, preserving function in a rat model of MI [291–293]. Similarly, intravenous or intramyocardial injection of apoptotic peripheral blood mononuclear cells significantly reduced infarct size related to the proportion of elastin to collagen [294]. Preservation of elastin likely protects heart biomechanics by reducing compensatory fibrotic remodeling processes and presents a novel therapeutic target to prevent eventual heart failure due to fibrosis and dilation post-MI. Additionally, transfection of *ex-vivo* porcine skin, EA. hy926 cells, human fibroblasts, and mesenchymal stem cells with synthetic tropoelastin mRNA subsequently increased insoluble elastin matrix deposition [291–293,295]. Stimulation of VSMCs isolated from mouse models of female pelvic floor disorder or aortic aneurysm with TGF-β and hyaluronan oligomers increased synthesis and deposition of extracellular elastin matrix [296–299]. Similarly, addition of Cu^2+^ ions increased elastin deposition through enhancing LOX activity [300].

**Fig. 4.**
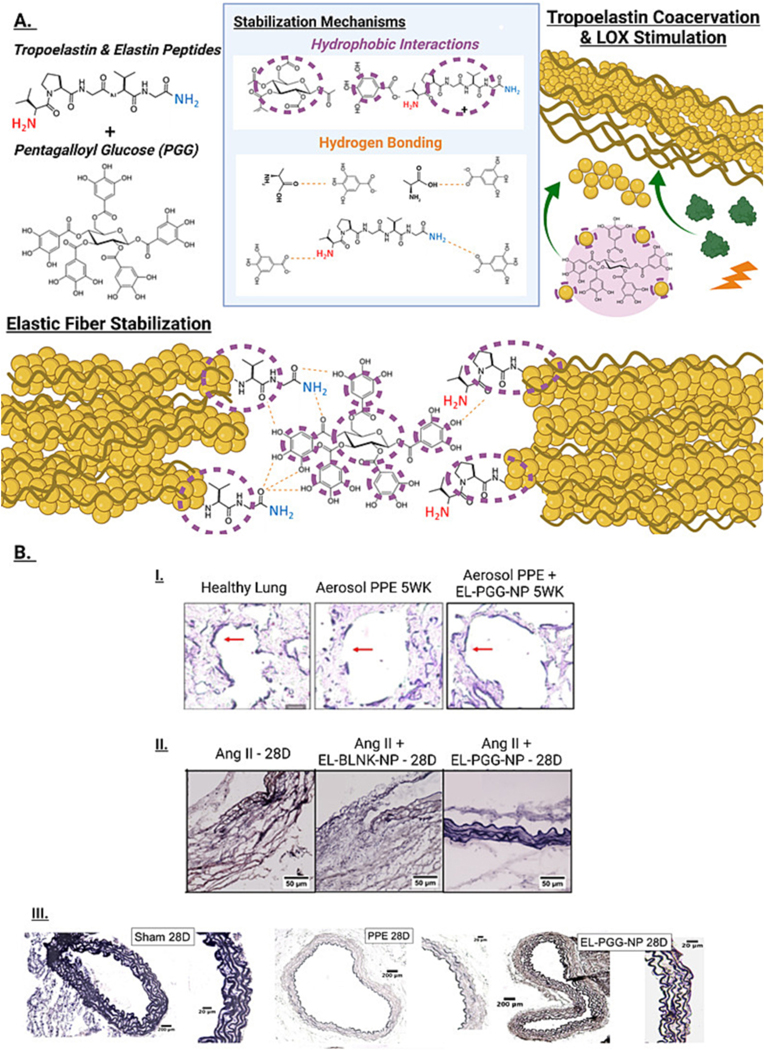
PGG-Elastin Interactions: A) Due to hydrophobic nature, PGG binds to hydrophobic domains in elastic fibers and tropoelastin and can also form hydrogen bonds; B) Systemic therapy with degraded elastin targeting nanoparticles loaded with pentagalloyl glucose (EL-PGG-NPs) restores elastic fibers in murine disease models. (I) Luna staining of lungs in porcine pancreatic elastase (PPE) aerosol induced emphysema after treatment with EL-PGG-NPs shows stabilization of the elastic layer of the alveolar wall [311]. (II) Verhoef van Gieson (VVG) staining of abdominal aorta after aneurysm development via Angiotensin II infusion showing elastin degradation, EL-PGG-NPs therapy reverses aneurysms and restores elastin in the medial layer [312]. (III) VVG staining of abdominal aorta elastase-induced aneurysm model. Elastin degradation 28 days after aneurysm development (PPE 28D), EL-PGG-NPs therapy reverses aneurysms and restores elastin in the medial layer [315].

Given the lack of therapies currently available for elastin-related pathologies, research in our laboratory over the last two decades has focused on stabilizing elastic fibers and restoring lost elastin. Elastin can be stabilized with polyphenols such as ellagic acid, tannic acid, and pentagalloyl glucose (PGG), which occurs by various mechanisms described in Fig. 4 [301,302]. PGG and tannic acid treatment is beneficial in preserving vascular elastin from elastase; application of PGG to aneurysmal aortas prevents further degradation of elastin and reduces the size of aneurysms [303,304]. Polyphenols not only stabilize elastic fibers, but they also increase insoluble elastin production. PGG, epigallocatechin gallate, and catechin were shown to increase coacervation of tropoelastin monomers as well as enhance LOX activity causing formation of mature elastic fibers from rat pulmonary fibroblasts as well as aortic VSMCs. Additionally, polyphenol treatments decreased cellular elastolytic MMP-2 and MMP-9 activity, which can further prevent the degradation of elastin [302,305]. Elastic fiber bound PGG can bind cell secreted tropoelastin to anchor it in place, thus despite reduction of elastic fiber assembly in adults, the combined effects of PGG can facilitate regeneration of damaged mature elastin. Specifically in aged mice, oral administration of polyphenol containing dill extract was capable of stimulating neo synthesis of elastic fibers associated with the reversal of cardiac hypertrophy and thoracic aortic dilation [306].

Dietary polyphenol intake has been associated with reduction of elastin related disease such as AA. In both rat and mouse models of CaCl_2_/elastase induced AA oral administration of polyphenols reduced the incidence of AA associated with increases in tropoelastin and LOX expression as well as suppression of MMP-2 and 9 activity [307,308]. Despite this, orally ingested polyphenols undergo phase II metabolism and rapidly degrade in the intestine, liver, and kidneys greatly reducing their effective to administered dose [309]. Thus, targeted therapy can be used to deliver polyphenols at a greater active dose to the site of elastin degradation. We previously demonstrated intravenous application of nanoparticles conjugated with antibodies targeting degraded elastin (EL-NPs) localize them to the fragmented elastin in emphysematous lungs, abdominal AAs, and calcified arteries [310–312]. Treatment with PGG-loaded EL-NPs significantly reverses elastin damage in murine models of vascular calcification, AAA, and emphysema/chronic obstructive pulmonary disease, resulting in reduced pathology – indicating the importance of elastin stability [312,313]. EL-PGG-NP treatment partially restored the mechanical properties of murine emphysematous lungs and aneurysmal abdominal aortas, with reduced inflammatory macrophages, T cells, and MMP expression in AA models [311,312,314,315]. As seen in Fig. 4, diseased lung alveolar walls and aneurysmal aortic tissues lack elastic layers, and they are restored after treatment with EL-PGG-NPs. Fragmented elastic lamina in aneurysmal aorta was also restored after EL-PGG-NP therapy in two rodent models (Fig. 4).

Interestingly, elastin stabilization by EL-PGG-NPs in aortic aneurysms also correlated with reduced TGF-β in aneurysmal tissues implying that PGG mediated restoration of elastic fiber may halt cellular TGF-β secretion or prevent release from LTBPs in elastin network [315]. While TGF-β and associated signaling activity are increased in aged arterial walls as well as animal models of and human patients with Marfan syndrome, systemic TGF-β neutralization in murine AAA models results in increased aneurysmal growth and mortality due to rupture [47,316–318]. Loeys-Dietz Syndrome patients are predisposed to developing Kawasaki Disease, an acute inflammatory disease of unknown etiology which includes coronary artery aneurysm formation and aortic root dilatation [319]. Like AA models, in mouse models of Kawasaki Disease, inhibition of TGF-β caused increased MMP-9 mediated elastin degradation and worsened vascular lesions [320]. Further complicating these effects, increased polyphenol induced LOX can bind TGF-β suppressing signaling, while systemic inhibition of LOX using beta-aminopropionitrile induces aortic aneurysm, rupture, and increases aberrant TGF-β signaling [321,322]. Subsequently, the effect of polyphenols on TGF-β as well as LOX and their role in vascular pathology requires further investigation. Overall, the ability of polyphenols to stabilize existing elastin structure, promote deposition of new elastic matrix by tropoelastin coacervation and LOX stimulation, and prevent inflammation presents a unique strategy to treat diseases where degradation of elastin is a crucial factor in disease pathology.

## Figures and Tables

**Table 1:** Genetic diseases that cause elastic fiber malformations and degradation.

Hereditary syndrome	Mutated gene	Affected protein domains	Mutation type	Clinical manifestations of disease related to elastin degradation	References
Alpha anti-trypsin deficiency	SERPINA1	Shutter domain	Loss of function/hypomorphic (activity)	Aortic dilation, vascular stiffness, emphysema	([48], [49])
Arterial tortuosity syndrome	SLC2A10	Trans-membrane domains - disruption of secondary structure	Loss of function/hypomorphic (activity)	Lax skin, arterial tortuosity	[50]
Autosomal Dominant Cutis Laxa (ADCL)	Type I-III: ELN, FBLN5, ALDH18A1	ELN - extended carboxy-terminal missense peptide sequence, FBLN5 - calcium-binding epidermal growth factor-like domain ALDH18A1 - gamma-glutamyl kinase domain	Hypomorphic (structure/expression)	Lax skin, aortic aneurysm, emphysema	[51,52]
Autosomal Recessive Cutis Laxa Type 1 (ARCL1)	Subtype A-C: FBLN5, FBLN4, LTBP4	FBLN5 - calcium-binding epidermal growth factor-like domain FBLN4 - N-terminal calcium-binding consensus sequence LTBP4 - fibulin 4/5 and tropoelastin binding domains	Hypomorphic (structure)	Aortic aneurysm, lax skin, emphysema, arterial tortuosity, gastrointestinal disfigurement	[53–55]
Autosomal Recessive Cutis Laxa Type 2 (ARCL2)	ATP6VOA2	Premature stop codon resulting in non-functional protein N-terminal cytoplasmic portion and trans-membrane domains	Loss of function/hypomorphic (activity/expression)	Wrinkly skin	[56]
Autosomal Recessive Cutis Laxa Type 3 (ARCL3)	Subtype A&B: ALDH18A1, PYCR1	ALDH18A1 - G5K/G5PR domain PYCR1 - reductase functional domain-coding region	Loss of function/hypomorphic (activity/expression)	Cataracts, wrinkled skin, and joint laxity	[57,58]
Beals-Hecht Syndrome	FBN2	Calcium-binding epidermal growth factor-like domain	Hypomorphic (structure)	Movement limitations and extended limbs	[59]
Geleophysic Dysplasia	Type 1–3: ADAMTSL2, FBN1, LTBP3	ADAMTSL2 - TGFβ-binding protein-like domain-5 FBN1 - TGFβ-binding protein-like domain 5 LTBP3 - calcium-binding epidermal growth factor-like domain	Hypomorphic (structure)	Stiff joints, thickened skin, heart valve defects	[60,61]
GM1 Gangliosidosis	GLB1	β-Galactosidase catalytic domain	Loss of function/hypomorphic (activity)	Cardiomyopathy, connective tissue defects	[62]
Hurler disease	IDUA	Immunoglobulin-like domain	Loss of function	Connective/skeletal/heart valve defects, cardiomyopathy, coronary artery stenosis	[63]
Isolated Ectopia Lentis (ECTOL)	Type 1&2: FBN1, ADAMTSL4	FBN1 - calcium-binding epidermal growth factor-like domain	Loss of function/hypomorphic (structure)	Stiff joints, lens dislocation, visual impairment	[64,65]
Loeys-Dietz Syndrome (LDS)	Type I-IV: TGF-BR1, TGF-BR2, SMAD3, TGF-B2	TGF-BR1 - Kinase Domain TGF-BR2 - Kinase Domain SMAD3 - MH2 domain TGF-B2 - Assorted domain nonsense/frameshift mutations	Loss of function/hypomorphic (activity)	Aortic aneurysm, lax skin, extended extremities	[66]
Marfan Syndrome (MFS)	FBN1	Calcium-binding epidermal growth factor-like domain	Hypomorphic (structure)	Aortic aneurysm, lax skin, extended extremities	[67]
MASS Syndrome	FBN1	Calcium-binding epidermal growth factor-like domain	Hypomorphic (structure)	Lax skin, mitral valve prolapse	[68]
Menkes Disease	ATP7A	Trans-membrane and catalytic domains	Loss of function	Blindness, respiratory failure, lax skin, kinked hair	[69]
Morquio Disease	Type A&B: GALNS, GLB1	GALNS - hydrophobic core region GLB1 - β-galactosidase catalytic domain	Loss of function/hypomorphic (activity/expression)	Valvular disease, cardiac hypertrophy, inguinal hernia, corneal clouding, joint laxity, small but normal functioning lungs	[70,71]
Occipital Horn Syndrome (OHS)	ATP7A	ATP-binding domain	Hypomorphic (activity)	Loose skin and joints, development of occipital bone spurs	[72]
Pseudoxanthoma Elasticum	ABCC6	C-terminal cytoplasmic loop, C-terminal nucleotide-binding domain	Loss of function	Yellow dermal plaques, visual impairment, vascular calcification, hypertension, myocardial infarction	[73]
Shprintzen–Goldberg Syndrome (SGS)	SKI	SMAD-binding domain	Hypermorphic (activity)	Valvular disease, aortic root dilation/aortic aneurysm, respiratory distress, abdominal hernias	[74]
Sorsby Fundus Dystrophy (SFD)	TIMP3	C-terminal domain	Hypomorphic (activity)	Visual impairment, chorodial neovascularization	[75]
Stiff skin syndrome (congenital scleroderma)	FBN1	Calcium-binding epidermal growth factor-like domain	Hypomorphic (structure)	Abnormally stiff skin and joints	[76]
Weill-Marchesani syndrome (WMS)	Type 1–4: ADAMTS10, FBN1, LTBP2, ADAMTS17	ADAMTS10 - premature stop codon in metalloprotease domain FBN1 - LTBP binding domain LTBP2 - Calcium-binding epidermal growth factor-like domain ADAMTS17 - C-terminal thrombospondin repeats	Hypomorphic (activity, structure)	Stiff joints, thickened skin, ocular lens dislocations, heart valve defects	[77–80]
William’s Beuren Syndrome (WBS)	ELN	Complete deletion on single chromosome	Hypomorphic (expression)	Supravalvular aortic stenosis, arterial lesions, heart disease, inguinal hernia, stroke	[81]

**Table 2:** Clinical manifestation of elastic fiber degradation in different organs.

Organ system	Clinical manifestations of disease related to elastic fiber degradation
Cardiovascular, hepatic, and renal	Vascular aneurysms (aortic, venous, coronary), atherosclerosis, myocardial infarction, vascular calcification, diabetes, non-alcoholic fatty liver disease, non-alcoholic steatohepatitis, chronic kidney disease, heart valve defects, parasitic/viral infection
Dense connective tissue	Hernia, lax or stiff joints and tendons, pelvic floor prolapse, Peyronie’s disease, floppy eyelid disease, Dupuytren’s contracture
Dermal	Lax or stiff skin, dermal plaques or lesions
Opthalmic	Age-related macular degeneration, pseudoexfoliation syndrome, diabetic retinopathy, rhegmatogenous retinal detachment, abnormal eye geometry, glaucoma
Pulmonary	Emphysema, chronic obstructive pulmonary dIsorder, idiopathic pulmonary fibrosis, microbial/viral Infection

## Data Availability

No data was used for the research described in the article.

## Glossary

AA: aortic aneurysm
CKD: chronic kidney disease
EBP: elastin binding protein
ECM: extracellular matrix
EDPs: elastin derived peptides
Eln: gene for tropoelastin
EL-(PGG)-NP: elastin targeted – (PGG loaded) - nanoparticle
VSMCs: vascular smooth muscle cells
LPS: lipopolysaccharide
LOX: lysl oxidase
LTBP: latent transforming growth factor β binding protein
MMP: matrix metalloprotease
MI: myocardial infarction
PGG: pentagalloyl glucose
TGF-β: transforming growth factor β
TIMP: tissue inhibitor of metalloproteinase
